# Patients’ ability to treat anaphylaxis using adrenaline autoinjectors: a randomized controlled trial

**DOI:** 10.1111/all.12628

**Published:** 2015-04-16

**Authors:** T. Umasunthar, A. Procktor, M. Hodes, J. G. Smith, C. Gore, H. E. Cox, T. Marrs, H. Hanna, K. Phillips, C. Pinto, P. J. Turner, J. O. Warner, R. J. Boyle

**Affiliations:** ^1^ Section of Paediatrics Imperial College London London UK; ^2^ Imperial College Healthcare NHS Trust St. Mary's Hospital London UK; ^3^ Academic Unit of Child and Adolescent Psychiatry Imperial College London London UK; ^4^ Population Health Research Institute St. George's, University of London London UK; ^5^ Department of Paediatric Allergy Division of Asthma, Allergy and Lung Biology King's College London London UK

**Keywords:** adrenaline, anaphylaxis, autoinjector, food allergy, human factors research

## Abstract

**Background:**

Previous work has shown patients commonly misuse adrenaline autoinjectors (AAI). It is unclear whether this is due to inadequate training, or poor device design. We undertook a prospective randomized controlled trial to evaluate ability to administer adrenaline using different AAI devices.

**Methods:**

We allocated mothers of food‐allergic children prescribed an AAI for the first time to Anapen or EpiPen using a computer‐generated randomization list, with optimal training according to manufacturer's instructions. After one year, participants were randomly allocated a new device (EpiPen, Anapen, new EpiPen, JEXT or Auvi‐Q), without device‐specific training. We assessed ability to deliver adrenaline using their AAI in a simulated anaphylaxis scenario six weeks and one year after initial training, and following device switch. Primary outcome was successful adrenaline administration at six weeks, assessed by an independent expert. Secondary outcomes were success at one year, success after switching device, and adverse events.

**Results:**

We randomized 158 participants. At six weeks, 30 of 71 (42%) participants allocated to Anapen and 31 of 73 (43%) participants allocated to EpiPen were successful – RR 1.00 (95% CI 0.68–1.46). Success rates at one year were also similar, but digital injection was more common at one year with EpiPen (8/59, 14%) than Anapen (0/51, 0%, *P* = 0.007). When switched to a new device without specific training, success rates were higher with Auvi‐Q (26/28, 93%) than other devices (39/80, 49%; *P* < 0.001).

**Conclusions:**

AAI device design is a major determinant of successful adrenaline administration. Success rates were low with several devices, but were high using the audio‐prompt device Auvi‐Q.

Anaphylaxis is life‐threatening and increasingly reported 1, 2. Prompt use of an adrenaline autoinjector (AAI) is the first‐line community treatment for anaphylaxis, and AAI sales have increased in parallel with anaphylaxis hospitalizations 1, 3. As emergency medical devices, AAIs should be simple to use; however, cross‐sectional surveys suggest that patients, their parents and even healthcare practitioners are unable to use commonly prescribed AAIs 4, 5, 6, 7. Indeed, reports of accidental digital injection using an AAI are common 8. These findings may be due to a lack of adequate training, or to inherent flaws in AAI device design. Prospective studies are required to establish whether AAI device design is adequate, and whether there are important differences between AAIs that impact on ability to successfully administer adrenaline for anaphylaxis. We undertook a prospective randomized controlled trial to evaluate the ability of mothers of food‐allergic children, prescribed and trained with an AAI for the first time, to successfully administer adrenaline in a simulated anaphylaxis scenario. We compared success rates in mothers allocated to different AAI device designs.

## Materials and methods

### Study design

We conducted a two‐by‐two factorial randomized controlled trial to investigate the effect of adrenaline autoinjector (AAI) device design on participants' ability to administer adrenaline in a simulated anaphylaxis scenario six weeks (primary outcome) and one year after training, in mothers of food‐allergic children. Fathers or other carers were not included in this study. After completion of the one‐year assessment, we then randomly reallocated participants to a further simulated anaphylaxis scenario using one of five different AAI device designs, without specific training in the new AAI device, to assess the safety of switching devices without further device‐specific training. Here, we report the outcomes of the AAI trial main study (ISRCTN12504076) and the AAI device switch study (ISRCTN29175528). We also simultaneously evaluated a psychological intervention for reducing maternal anxiety, which will be reported elsewhere.

### Participants

#### Main study

Mothers of children aged 0–18, diagnosed with food allergy by a paediatric allergist, and deemed to need an AAI as part of their food allergy management were eligible for inclusion. Exclusion criteria were inability to converse fluently in English or with a translator, child weight <7.5 kg, prior training in an AAI device, and significant psychiatric problems such as psychotic disorders.

#### Device switch study

Main Study participants who completed the outcome assessment at one year (±3 months) were eligible for inclusion in the Device Switch Study. Exclusion criteria were training on an alternative AAI device between randomization for the Main Study and the one‐year visit, and significant psychological distress at the one‐year scenario.

### Study setting

Participants were recruited from a large specialist paediatric allergy centre in an urban setting in London, UK, between March 2011 and December 2012. Researchers invited mothers of children with food allergy who were being prescribed an AAI for the first time to participate. Written informed consent was obtained prior to participation in any study procedures, and the study was approved by the West London Research Ethics Committee (10/H0711/76).

### Randomization, treatment allocation and masking

(i). First randomization (‘Main Study’). Treatment was allocated using a computer‐generated randomization list in blocks of four, stratified by maternal anxiety (State Trait Anxiety Inventory) score, generated by an independent statistician (Imperial College London Statistical Advisory Service). The randomization list was held by a clinical trials pharmacist – researchers notified the pharmacist after enrolling a participant, and the pharmacist allocated treatment and dispensed an AAI based on the randomization list.

(ii). Second randomization (‘Device Switch Study’). Treatment was allocated using a computer‐generated randomization list stratified by ‘Main Study’ treatment allocation (Anapen or EpiPen) and by success in AAI administration at one year. The randomization list was generated by an independent statistician (Imperial College London Statistical Advisory Service) and held by a clinical trials pharmacist. Researchers notified the pharmacist after enrolling a participant, and the pharmacist allocated a new AAI device for the anaphylaxis scenario based on the randomization list.

It was not possible to mask clinicians, participants or outcome assessors to AAI treatment allocation. To reduce ascertainment bias, anaphylaxis scenario assessments were video recorded and scored by a paediatric allergist independent of the study sponsor, institution or investigators (TM).

### Intervention

Main Study: participants were randomized to receive Anapen^™^ (Lincoln Medical, London, UK) or an EpiPen^™^ (Mylan, Basking Ridge, NJ, USA) with standardized training. Information included recognition of anaphylactic reactions, management of such reactions including how and when to use their AAI, and provision of a trainer AAI. The devices are shown in Fig. S1. A researcher gave a practical demonstration of the use of the device and ensured participants were able to demonstrate correct device technique before leaving the training session. Written device‐specific information was provided, which was approved by the relevant device manufacturer. All research staff were trained in anaphylaxis management through a national training programme (Allergywise, Anaphylaxis Campaign, UK).

Device Switch Study: participants were randomly allocated to be assessed using one of five alternative trainer AAI devices. These were a new EpiPen device design (Mylan), released during the course of the trial, JEXT^™^ (ALK‐Abello, Horsholm, Denmark), Anapen/EpiPen (whichever device the participant had not been trained on during the Main Study), or a device with audio prompts Auvi‐Q^™^ (Sanofi US, Kansas City, MO, USA). The devices New EpiPen, JEXT and Auvi‐Q are shown in Fig. S2. Participants did not receive any specific training with the new device, but were asked to use it in a simulated anaphylaxis scenario.

### Outcome measures

Primary outcome measure was successful adrenaline administration using a trainer AAI during a simulated anaphylaxis scenario, six weeks after being trained for the first time (Main Study). The four key steps needed for success were as follows: removal of all safety caps, placement of correct end of the device against the thigh, activation of device and holding device in place for adrenaline delivery for ≥5 s. Where participants failed to deliver adrenaline, primary reason for failure was defined using a hierarchy of ‘Safety cap(s) removal > correct device positioning > device activation > held in place for sufficient time’. The first step failed is the primary reason for failure. Secondary outcomes included successful administration based on the same four key steps, but using the adrenaline discharge time reported by the AAI manufacturer (3.2 s Anapen, 1 s all other devices) instead of 5 s; successful administration of adrenaline at one year; success after switching device; adverse events at each anaphylaxis scenario (digital injection) and participants’ confidence in how to use the device at six weeks and one year (Main Study). The independent allergist assessed participant actions using video recordings, with a device‐specific scoring sheet, to decide whether adrenaline would have been successfully delivered using 5‐s and device‐specific criteria. Full details of the simulated anaphylaxis scenario are described in the Appendix S1.

### Salivary stress response to simulated anaphylaxis

We also evaluate salivary cortisol and α‐amylase levels in participants before and after the one‐year simulated anaphylaxis scenario (Main Study). For this analysis, samples were taken immediately prior to the scenario, and 10, 20 and 30 min following the scenario. Saliva samples were collected using cotton swabs (Salivettes; Sarstedt, Numbrecht, Germany) and stored at −20°C until analysis. Salivary cortisol and α‐amylase levels were measured by a commercial company using published methodology 9.

### Changes to study design

Anapen AAI (Lincoln Medical) was withdrawn from the UK market on 23 May 2012. Those participants enrolled subsequently (*n* = 42) were therefore not randomly allocated to an AAI device– all were allocated to EpiPen (Mylan). Participants who had already been allocated to Anapen were invited for a six‐week or one‐year (± Device Switch) outcome assessment as appropriate.

### Statistical methods

#### Sample size calculation

Sample size for the Main Study was calculated on the basis that 63% of participants would successfully deliver adrenaline six weeks after training 6. With 86 participants in each arm, we had 80% power at 5% two‐sided significance using chi‐squared test to detect whether Anapen training results in successful adrenaline delivery in 83% *vs* 63% for EpiPen. Assuming 15% loss to follow‐up at six weeks, we planned to enrol 200 participants. Because of the unexpected withdrawal of Anapen from the UK market part‐way through the study, we were able to recruit 79 patients in each arm (total 158). Assuming 80% of Main Study participants are assessed at one year, and 80% of these agree to participate in a second anaphylaxis scenario (Device Switch Study), we planned to undertake 128 s randomizations to undergo assessment with a new AAI device (32 Auvi‐Q; 32 New EpiPen; 32 JEXT; 32 Alternative device, i.e. Anapen/EpiPen). With 128 paired comparisons, assuming a 50% success rate in the Main Study one‐year anaphylaxis scenario, the Device Switch Study had 80% power at 2‐sided alpha 0.01 to detect whether 20% of participants succeed in the Main Study one‐year assessment and fail in the Device Switch assessment, compared with 5% failing in the Main Study one‐year assessment/succeeding in Device Switch.

#### Outcome analysis

We used chi‐squared or Fisher's exact test, and logistic regression for adjusted analyses of binary outcomes, to calculate odds ratios (OR) and 95% confidence intervals (95% CI). We used Mann–Whitney *U*‐test for continuous data because they were non‐normal as per Kolmogorov–Smirnov test. We used bootstrapping to estimate 95% CI around mean differences, and McNemar mid‐p test for binary matched‐pair data 10. Primary analysis was conducted with intention‐to‐treat population and secondary analysis with per‐protocol (PP) population. Assessment of successful adrenaline delivery and adverse events was also made contemporaneously by the researcher. Due to good agreement between researcher and independent assessor (TM) judgments (kappa score for successful adrenaline delivery 0.88, six weeks; 0.89, one year; 0.95, Device Switch Study), where the primary outcome measure could not be determined from the video recording, contemporaneous researcher assessment was used (*n* = 9 cases for primary outcome assessment). PP analysis at six weeks included participants nonrandomly allocated to EpiPen, and excluded those who did not receive intervention, did not attend six‐week follow‐up within 90 days of treatment allocation, and whose outcomes were based on contemporaneous researcher assessment. PP analysis at one year also included participants nonrandomly allocated to EpiPen, or not seen at one year (±3 months), and whose outcomes were based on contemporaneous researcher assessment. All statistical analyses used SPSS v21.0 (IBM, Armonk, NY, USA).

## Results

**Figure 1:**
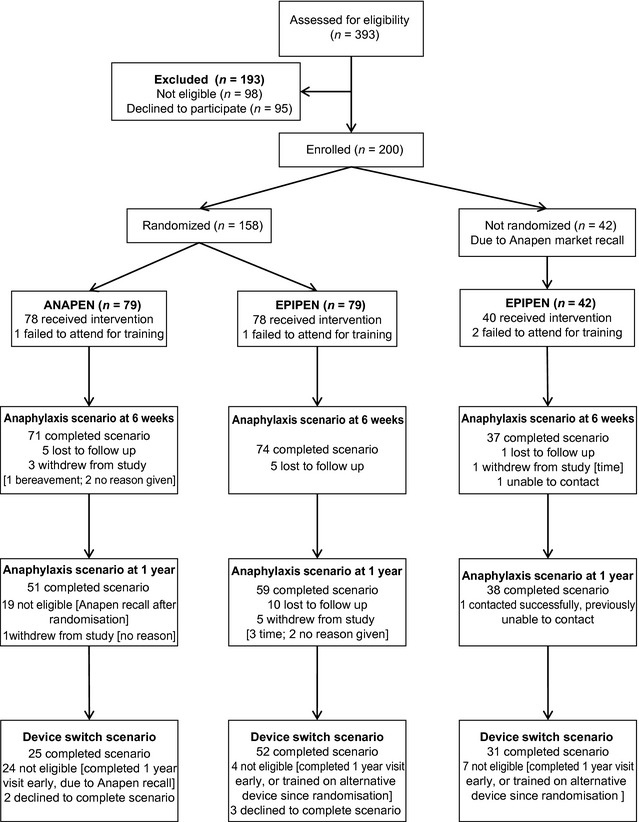
CONSORT flow diagram.

We enrolled 158 randomized participants between 8 March 2011 and 23 May 2012. The 42 participants enrolled after 23 May 2012 could not be allocated to Anapen due to recall of Anapen by the UK National Regulator, resulting in withdrawal of Anapen from the UK market. These participants were therefore nonrandomly allocated to EpiPen. The last such participant was enrolled on 10 December 2012. Participant flow is shown in Fig. 1. Overall, 182 (91%) participants and 145 (92%) randomized participants were assessed for the primary outcome at six weeks, and 148 (74%) and 110 (70%) at one year. Characteristics of study participants at enrolment are shown in Table 1. The randomized groups were similar, but there was a difference in personnel administering AAI training in those participants nonrandomly allocated to EpiPen, due to a staffing change part‐way through the trial, and improved asthma control in the nonrandomized group. Those participants who failed to complete the study at one year were more commonly not living with their partner, had a nonprofessional occupation, or were of nonwhite ethnicity than those who completed the study; noncompleters also more commonly had a child >25 kg (Tables S1 and S2).

**Table 1:** Participant characteristics at baseline

	Anapen (79)	EpiPen (79)	*P*	Not randomized EpiPen (42)	*P*
Maternal age (years)	36.4 (6.4)	35.6 (6.2)	0.48	36.2 (6.0)	0.86
Age left full‐time education (years)	22.7 (5.3)	23.0 (6.3)	0.95	22.2 (3.8)	0.84
Living with partner	61 (79.2)	60 (76.9)	0.73	28 (75.7)	0.75
Professional occupation	37 (57.8)	35 (50.7)	0.41	17 (54.8)	0.94
Worked in a healthcare setting	11 (16.2)	17 (25.0)	0.20	8 (22.9)	0.77
Nonwhite ethnicity	48 (63.2)	46 (59.0)	0.60	22 (57.9)	0.72
Number of children in household					
One child	31 (39.7)	31 (40.8)		23 (60.5)	
Two children	33 (42.3)	27 (35.5)		11 (28.9)	
Three or more children	14 (17.9)	18 (23.7)	0.58	4 (10.5)	0.07
Maternal state anxiety [STAI‐1]	36.1 (11.5)	36.3 (11.3)	0.84	37.4 (12.6)	0.64
Child age (years)	4.5 (3.7)	3.5 (2.9)	0.06	3.8 (3.2)	0.88
Male child	47 (59.5)	50 (63.3)		25 (59.5)	
Child weight ≥25 kg	18 (22.5)	12 (15.2)	0.22	6 (14.6)	0.52
No food allergies	2.6 (1.6)	3.2 (2.1)	0.05	2.4 (1.3)	0.17
Eczema in child	56 (75.7)	65 (86.7)	0.08	30 (76.9)	0.55
Eczema severity [POEM]	9.9 (7.3)	9.8 (7.7)	0.80	8.7 (5.5)	0.70
Asthma in child	21 (28.4)	18 (24.0)	0.54	5 (12.8)	0.08
Partially/Uncontrolled asthma	17 (23.0)	14 (18.7)	0.55	2 (5.1)	0.02
Allergic rhinitis in child	23 (31.5)	20 (26.7)	0.52	14 (36.8)	0.35
Moderate/Severe allergic rhinitis	21 (28.8)	18 (24.0)	0.51	12 (31.6)	0.52
History of anaphylaxis^a^	27 (35.1)	25 (32.1)	0.70	9 (23.1)	0.21
Anaphylaxis training officer					
Researcher #1	13 (16.7)	13 (16.5)		0 (0.0)	
Researcher #2	29 (37.2)	26 (32.9)		20 (51.3)	
Researcher #3	36 (46.2)	40 (50.6)		0 (0.0)	
Researcher #4	0 (0.0)	0 (0.0)	0.83	19 (48.7)	<0.001
Randomized to psychological intervention	40 (50.6)	39 (49.4)	0.87	22 (52.4)	0.78
Days since training at primary outcome assessment	55.4 (17.7)	53.7 (19.4)	0.64	57.0 (19.7)	0.38

Continuous data are presented as mean (SD), categorical data as *n* (%). STAI, state trait anxiety inventory; POEM, patient‐oriented eczema measure.

a Anaphylaxis was defined according to NIH/NIAID guidance (2).

### Effect of AAI design on successful administration in a simulated anaphylaxis scenario

**Figure 2:**
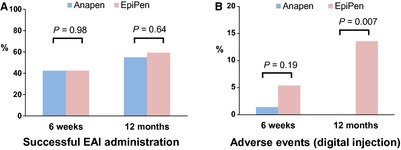
Main Study Outcomes. Rates of successful adrenaline administration (A) and digital injection (B) in participants randomly allocated to Anapen or EpiPen at 6 weeks and one year following initial training.

The primary outcome successful AAI administration did not differ between Anapen and EpiPen groups at six weeks or one year, when either 5 s or device‐specific delivery times were used to assess success (Table 2; Fig. 2A). Participants who completed the one‐year visit were more likely to have been successful at six weeks (Table S3). Imputation for missing data did not change the study findings (Table S4). There was no significant difference between groups in the primary reason for failure at six weeks, but there was a significant difference at one year where the Anapen group more commonly failed to remove all safety caps, and the EpiPen group more commonly used the incorrect end of the device. At one year (but not at six weeks), the EpiPen group also had increased frequency of digital injection (Fig. 2B). Similar findings were seen in respect of primary reason for failure and digital injections in PP analysis. In PP analysis, there was also increased overall success in the EpiPen group when using device‐specific delivery times (but not a 5‐second delivery time) and EpiPen was held in place for longer than Anapen at six weeks, but these differences were not significant in adjusted analyses (Table S5).

**Table 2:** Ability to use an adrenaline autoinjector in participants randomly allocated to Anapen or EpiPen

	Anapen (79)	EpiPen (79)	*P*	RR (95% CI)
Primary outcome (six weeks)				
Successful AAI administration (5‐s criterion)	30 (42.3)	31 (42.5)	0.98	1.00 (0.68, 1.46)
Primary reason for failure				
Failed to remove all safety caps	26 (36.6)	21 (28.8)		
Used incorrect end of device	5 (7.0)	5 (6.8)		
Device not activated	0 (0.0)	3 (4.1)		
AAI applied for <5 s	10 (14.1)	13 (17.8)	0.27	
Secondary Outcomes (six weeks)				
Successful AAI administration (minimum discharge time)	32 (45.1)	40 (55.6)	0.21	0.81 (0.58, 1.13)
Adverse events (digital injection)	1 (1.4)	4 (5.4)	0.19	0.26 (0.03, 2.28)
Time device held in place (sec)	6.1 (4.7)	7.2 (5.1)	0.20	1.07 (−0.64, 2.77)
Postscenario confidence (1–10) in using AAI device	7.4 (2.6)	7.5 (2.4)	0.90	0.15 (−0.68, 0.94)
Area massaged after simulated injection	40 (56.3)	40 (54.1)	0.78	1.04 (0.78, 1.40)
Device applied to correct anatomical position	60 (84.5)	66 (89.2)	0.40	0.95 (0.83, 1.08)
Child held in correct position	48 (67.6)	51 (68.9)	0.87	0.98 (0.79, 1.23)
Emergency services called	57 (80.3)	54 (73.0)	0.30	1.10 (0.92, 1.32)
Secondary Outcomes (one year)				
Successful AAI administration (5‐s criterion)	28 (54.9)	35 (59.3)	0.64	0.93 (0.67, 1.28)
Primary reason for failure				
Failed to remove all safety caps	17 (33.3)	6 (10.2)		
Used incorrect end of device	0 (0.0)	10 (16.9)		
Device not activated	0 (0.0)	1 (1.7)		
AAI applied for <5 s	6 (11.8)	7 (11.9)	<0.001	
Successful AAI administration (minimum discharge time)	30 (58.8)	42 (71.2)	0.17	0.83 (0.62, 1.10)
Adverse events (digital injection)	0 (0.0)	8 (13.6)	0.007	–
Time device held in place (sec)	7.1 (4.4)	8.5 (4.4)	0.15	1.40 (−0.24, 3.08)
Postscenario confidence (1–10) in using AAI device	7.2 (2.1)	7.0 (2.6)	0.99	0.17 (−1.01, 0.73)
Area massaged after simulated injection	41 (80.4)	37 (62.7)	0.04	1.28 (1.01, 1.63)
Device applied to correct anatomical position	49 (96.1)	58 (98.3)	0.60	0.977 (0.92, 1.04)
Child held in correct position	43 (84.3)	47 (79.7)	0.53	1.06 (0.89, 1.26)
Emergency services called	44 (86.3)	54 (93.1)	0.34	0.93 (0.81, 1.06)

Continuous data are mean (SD) and mean difference (95% CI), categorical data as *n* (%). AAI, adrenaline autoinjector.

### Effect of AAI design on salivary stress response in a simulated anaphylaxis scenario

There was an increase in salivary α‐amylase (*P* < 0.001) but not salivary cortisol (*P* = 0.37) level following the simulated anaphylaxis scenario at the one‐year visit. The change in salivary stress hormone levels did not differ significantly between participants randomized to EpiPen *vs* Anapen for either α‐amylase (*P* = 0.34) or cortisol (*P* = 0.66).

### Effect of AAI design on successful administration after Device Switch without retraining

Successful AAI administration rates in the Device Switch Scenario were comparable between participants originally trained with EpiPen and those trained with Anapen using either 5 s (*P* = 0.34) or device‐specific delivery times (*P* = 0.84), and these success rates did not differ overall, from success rates using the device on which they had been trained (Table 3). For example, success rates for participants trained on EpiPen and switched to New EpiPen or JEXT were 28/42 (67%) using EpiPen and 30/42 (71%) using the new device (*P* = 0.51). However, successful AAI administration in the Device Switch Scenario differed according to the specific device participants were randomly allocated to (*P* < 0.001; Table S6). Participants allocated to Auvi‐Q had the highest success rate (26/28, 93% Auvi‐Q *vs* 39/80, 49% other devices; *P* < 0.001), whether they were previously trained on Anapen or EpiPen, and this was higher than their success rate in the preceding anaphylaxis scenario using the device they had been trained to use (93% *vs* 57%; *P* = 0.006).

**Table 3:** Ability to use a different adrenaline autoinjector in participants trained to use Anapen or EpiPen

	Prior Anapen training (25) *n* (%)			Prior EpiPen training (83) *n* (%)			Total (108) *n* (%)		
	Anapen	New device	*P*	EpiPen	New device	*P*	Original device	New device	*P*
Successful AAI administration (5‐s criterion)	14 (56.0)	13 (52.0)	0.79	54 (65.1)	52 (62.7)	0.74	68 (63.0)	65 (60.2)	0.67
Successful AAI administration (minimum discharge time)	14 (56.0)	16 (64.0)	0.55	65 (78.3)	55 (66.3)	0.08	79 (73.1)	71 (65.7)	0.22
Adverse events (digital injection)	0 (0.0)	3 (12.0)	–	9 (10.8)	0 (0.0)	–	9 (8.3)	3 (2.8)	0.60

**Figure 3:**
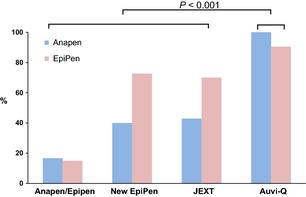
Device Switch Study Outcome. Rates of successful adrenaline administration in participants who had Anapen (blue) or old‐style EpiPen (red) for a year and were then randomly allocated to undergo an anaphylaxis scenario using a new device, without training.

There was a significant difference in success rate for participants switched to a 2‐cap device (Anapen) from a single‐cap device (EpiPen, New EpiPen, JEXT or Auvi‐Q), or vice versa (16/45, 36%), compared with participants switched between different single‐cap devices (49/63, 78%; *P* < 0.0001), and those switched between Anapen and EpiPen had the lowest success (15%; Fig. 3). This difference remained when Auvi‐Q was removed from analyses – 9/38 (24%) single‐cap/2‐cap switch, 30/42 (71%) single‐cap/single‐cap switch (*P* < 0.0001) – and was similar when device‐specific adrenaline delivery times were used (*P* < 0.0001; data not shown). Digital injection rates did not differ significantly between the 5 devices, but were increased in participants originally trained with Anapen when switched to a different device, compared with those originally trained with EpiPen (*P* = 0.01). The effect of a Device Switch in the community, with retraining, on successful AAI administration is described in the Appendix S1.

## Discussion

In this prospective randomized controlled trial in mothers of food‐allergic children, less than half of participants were able to administer adrenaline using a commonly prescribed AAI device in a simulated anaphylaxis scenario, six weeks following optimal training. When participants were switched to alternative but similar devices without retraining, success rates did not improve, but when switched to an audio‐prompt device Auvi‐Q, success rates were high. In contrast to previous work, our data clearly demonstrate that AAI device design, not simply AAI training, is critical for successful adrenaline delivery in an emergency scenario. Our data also highlight important areas for improvement in the design of some currently available AAI devices. This information is especially timely given the ongoing European Medicines Agency Section 31 review of AAI devices, triggered by the death of a young patient treated for anaphylaxis with an AAI [EMA/242569/2014 11].

Previous cross‐sectional surveys have found patients and healthcare practitioners are commonly unable to administer adrenaline using an AAI trainer 4, 5, 6, 7. However, such studies have not been able to determine whether the high failure rate is due to inadequate training or inherent issues with device design. In support of a role for training, a recent US study found that 16% of patients previously prescribed an AAI could perform all steps correctly, but success rates increased immediately after a formal training session, and this increase persisted 1 year later 12. Using a more rigorous simulated anaphylaxis scenario and prospective study design, we have clearly shown that device design is a critical factor in successful AAI use and that success rates using commonly prescribed devices are worryingly low even 6 weeks following training. In our study, all participants were optimally trained and were able to successfully administer adrenaline using a trainer AAI at the end of the initial training session. Failure to remove safety cap(s), use of the incorrect end of the device and holding the device in place for insufficient time were the most common primary reasons for failure, suggesting these are important areas for AAI device developers to focus on.

As the number of different AAI designs has increased, Device Switches have become a significant clinical issue. To our knowledge, this is the first study to directly address the safety of Device Switches. We found successful adrenaline administration rates were similar where the new device was similar to the old one. However, success rates dropped after switching between a one‐cap and a two‐cap device without training and increased after switching to the audio‐prompt device Auvi‐Q. These data suggest that care must be taken when a patient's AAI device is changed to an alternative design.

Strengths of our study include the prospective design in a representative clinic population, use of a simulated anaphylaxis scenario for assessments, which induced a significant stress response in participants, and outcome classification by an independent expert. AAI training was undertaken under optimal conditions, and all study participants demonstrated successful adrenaline administration using a trainer device prior to leaving their initial training session. Statistical power was limited by withdrawal of Anapen part‐way through the trial, meaning 21% of participants were nonrandomly allocated to an AAI device and some participants were not eligible for secondary outcome assessments. However, the study findings were clear‐cut, and robust in per‐protocol analysis and adjusted analysis and after imputation for missing data.

Our findings in a representative UK paediatric allergy clinic population may not be generalizable to other settings – in particular, the AAI training in some settings may be more limited than that received in the setting of a clinical trial, where staff were recently well trained, and participants were given as much time as they needed to ensure AAI training was complete and understood. For example, a recent survey of community pharmacists in Australia found that only 65% could correctly demonstrate to a ‘patient’ how to use their AAI 13. Mothers of food‐allergic children may have different performance characteristics in an emergency scenario to other patient groups at risk of anaphylaxis, so our findings cannot be generalized to all groups. Food allergy is however the commonest cause of anaphylaxis and is most prevalent among young children where mothers are often the primary caregiver, and fatal food anaphylaxis commonly occurs in the home environment – thus, mothers of food‐allergic children represent an important group for effective AAI training 1, 14. Finally, the availability of AAI devices varies considerably worldwide, with no AAIs marketed in some countries, and restricted choice of AAI in many areas. Our data will however inform treatment decisions in settings where more than one AAI device is available.

Our data provide the most conclusive evidence to date that there are significant design issues in some current AAI devices. Given that AAIs are associated with significant adverse events 8, that the evidence base for adrenaline in treating human anaphylaxis is weak 15, and that new noninjectable modes of adrenaline delivery may soon become available 16, we suggest that the role of different AAI device designs in clinical practice should be carefully evaluated within existing reviews of this area 11. In contrast, the high rate of successful adrenaline administration which we found when participants used the audio‐prompt device Auvi‐Q, in a stressful anaphylaxis scenario and without prior device‐specific training, suggests that Auvi‐Q is an important advance in AAI device design and may even be suitable for bystander use in public areas such as schools 17. Thus, Auvi‐Q or other audio‐prompt devices may play a similar role to automated external defibrillators with audiovisual cues, which are currently used for bystander treatment of cardiac arrest 18.

In summary, we have shown in a prospective randomized controlled trial that successful adrenaline administration rates using Anapen and EpiPen are low during simulated anaphylaxis, and EpiPen use under stressful conditions carries a significant risk of digital injection. Over 90% of participants were able to successfully administer adrenaline using Auvi‐Q without receiving device‐specific training, suggesting that AAI device design is critical to successful anaphylaxis management and this should be carefully assessed in the current European Medicines Agency AAI review.

## Funding

The authors were supported by the National Institute for Health Research (NIHR) Imperial Biomedical Research Centre, and the MRC‐Asthma UK Centre in Allergic Mechanisms of Asthma. P. J. Turner is in receipt of a Clinician Scientist award from the UK Medical Research Council. This study was supported by research grants from Lincoln Medical and the NIHR Comprehensive Research Network.

## Role of funders

The funders had no role in study design; in the collection, analysis, and interpretation of data; in the writing of the report; or in the decision to submit the article for publication.

## Role of researchers

The researchers are fully independent of the funding bodies which supported this trial. All authors, external and internal, had full access to all of the data (including statistical reports and tables) in the study and can take responsibility for the integrity of the data and the accuracy of the data analysis.

## Conflict of interest

All authors have completed the Unified Competing Interest form at www.icmje.org/coi_disclosure.pdf (available on request from the corresponding author) and declare that JOW is a trustee of the Anaphylaxis Campaign, and JOW and RJB have received research funding from the UK Food Standards Agency. The authors report no other relationships or activities that could appear to have influenced the submitted work.

## Author contributions

RJB conceived the study, secured funding for the trial and wrote the final version of the manuscript. JOW, CG, MH and HEC contributed to study design. TU wrote the study protocol and other clinical trials materials, supervised by RJB, secured regulatory approvals, set up the study and wrote the first draft of the manuscript. AP, HH, KP and CP recruited and trained participants and completed study assessments. TM performed independent assessments of videotaped simulated anaphylaxis scenarios for primary outcome evaluation. AP, RJB and JS wrote the statistical analysis plan. JS undertook all statistical analyses. All authors contributed to the interpretation of data analyses and drafting of the final manuscript.

## Supporting information




**Figure S1.** Epinephrine auto‐injector devices used in the Main Study.

**Figure S2.** Additional epinephrine auto‐injector devices used in the Device Switch Study.

**Appendix S1.** Supplementary Methods and Results.
**Table S1.** Participant characteristics at baseline, for randomised patients who did or did not complete outcome assessments at 6 weeks.
**Table S2.** Participant characteristics at baseline, for randomised patients who did or did not complete outcome assessments at one year.
**Table S3.** Six‐week outcomes for randomised patients who did or did not complete a one year outcome assessment.
**Table S4.** Results of imputation for missing data, for patients randomly allocated to Anapen or Epipen.
**Table S5.** Ability to use their epinephrine autoinjector in participants allocated to Anapen or Epipen – Per Protocol analysis.
**Table S6.** Ability to use different devices in participants trained to use Anapen or EpiPen.
