# Supplementary figures and images for: Patients’ ability to treat anaphylaxis using adrenaline autoinjectors: a randomized controlled trial
Source: Allergy. 2015 Apr 16;70(7):855–63. doi: 10.1111/all.12628 (PMC4654245; doi:10.1111/all.12628)

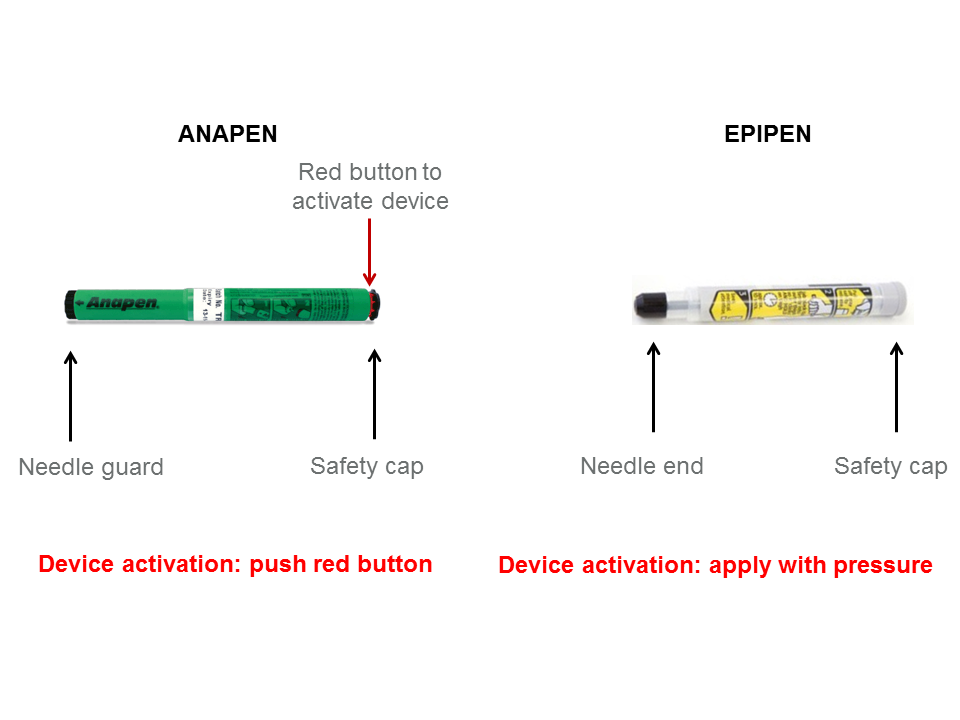

Supplement: Supplementary file 1 — Figure S1. Epinephrine auto‐injector devices used in the Main Study. [file ALL-70-855-s001.tif]

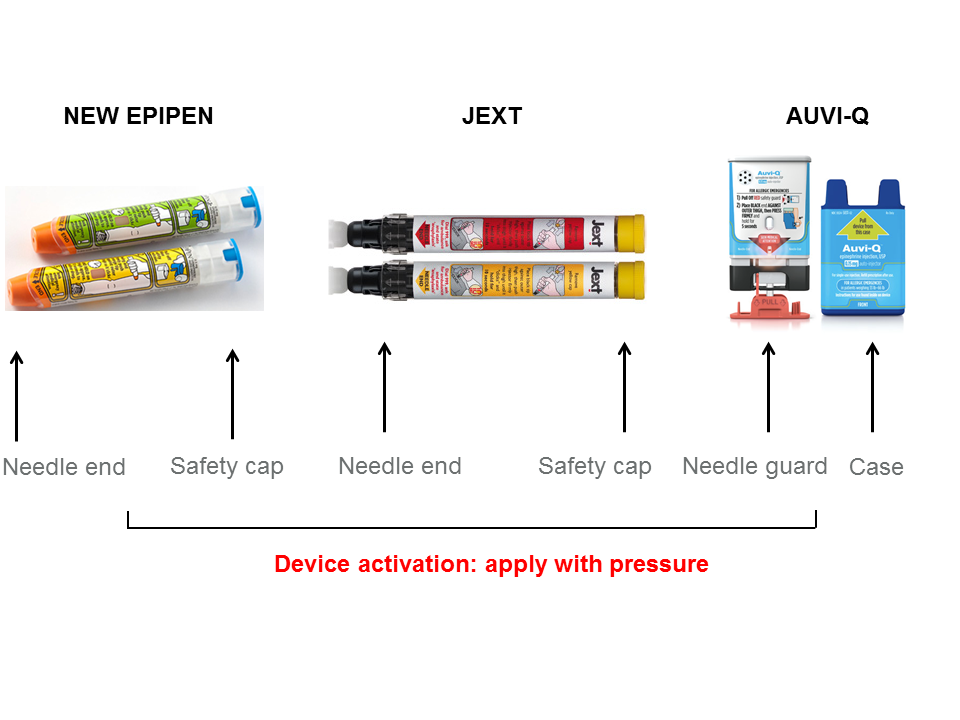

Supplement: Supplementary file 2 — Figure S2. Additional epinephrine auto‐injector devices used in the Device Switch Study. [file ALL-70-855-s002.tif]
