# Supplementary material for: Patients’ ability to treat anaphylaxis using adrenaline autoinjectors: a randomized controlled trial
Source: Allergy. 2015 Apr 16;70(7):855–63. doi: 10.1111/all.12628 (PMC4654245; doi:10.1111/all.12628)
Supplement: Supplementary file 3 — Appendix S1. Supplementary Methods and Results. Table S1. Participant characteristics at baseline, for randomised patients who did or did not complete outcome assessments at 6 weeks. Table S2. Participant characteristics at baseline, for randomised patients who did or did not complete outcome assessments at one year. Table S3. Six‐week outcomes for randomised patients who did or did not complete a one year outcome assessment. Table S4. Results of imputation for missing data, for patients randomly allocated to Anapen or Epipen. Table S5. Ability to use their epinephrine autoinjector in participants allocated to Anapen or Epipen – Per Protocol analysis. Table S6. Ability to use different devices in participants trained to use Anapen or EpiPen. [file ALL-70-855-s003.doc]

**Patients’ ability to treat anaphylaxis using Adrenaline Auto-injectors: a randomised controlled trial**

Thisanayagam Umasunthar^1, 2^, Annabella Procktor^1, 2^, Matthew Hodes^2, 3^, Jared G Smith^4^, Claudia Gore^1, 2^, Helen E. Cox^1, 2^, Tom Marrs^5, 6^, Heather Hanna^1, 2^, Katherine Phillips^1, 2^, Camila Pinto^1, 2^, Paul J. Turner^1^, John O. Warner^1, 2^, Robert J. Boyle^1, 2^

^1^ Section of Paediatrics, Imperial College London, Norfolk Place, London W2 1PG

^2^ Imperial College Healthcare NHS Trust, St. Mary’s Hospital, Praed Street, London W2 1PG

^3^ Academic Unit of Child and Adolescent Psychiatry, Imperial College London, Du Cane Road, London W12 0NN

^4^ Population Health Research Institute, St. George’s, University of London, Cranmer Terrace, London SW17 0RE

^5^ Department of Paediatric Allergy, Division of Asthma, Allergy and Lung Biology, King’s College London, SE1 7EH

**Address for Correspondence:**

Dr Robert Boyle

Section of Paediatrics

Imperial College London

Norfolk Place, London W2 1PG

United Kingdom

Tel: +44 207 594 3990; Fax: +44 207 594 3984; Email: r.boyle@imperial.ac.uk

**Key Words:** anaphylaxis; adrenaline; auto-injector; food allergy; human factors research

**METHODS**

**Study procedures**

Following randomisation and training, participants were invited for outcome assessment at six weeks and one year. If they failed to administer their AAI at six weeks they received repeat training following the anaphylaxis scenario. Prior to each scenario, confidence in using their device was assessed using a 10-point Likert scale. Participants were given a scenario where their child had developed clear cut anaphylaxis during a meal, with cutaneous signs of an allergic reaction and significant respiratory distress. An audio tape of a child wheezing was played, and participants were asked to demonstrate their management of the allergic reaction. A clothed dummy similar in size to their child was provided, a relevant AAI trainer device was modified so that all surface markings and instructions were as for a real device, and a telephone was placed close to the dummy so that participants could call an ambulance. Participants’ performance in simulated anaphylaxis scenarios was video recorded for evaluation by a pediatric allergist independent of the trial, funders and investigator’s institution (TM).

**RESULTS**

**Effect of community Device Switch with retraining, on successful AAI administration**

Forty-four participants underwent a Device Switch in the community during the course of the trial, due to withdrawal of Anapen from the UK market. These participants were then trained in EpiPen or JEXT, and 36 (82%) underwent a simulated anaphylaxis scenario using their new device 3 to 9 months after device switch. These participants thus constituted a separate group for the assessment of device switch safety - they were non-randomly allocated to switch from Anapen to an EpiPen or JEXT device, trained in the new device, and assessed in a simulated anaphylaxis scenario a mean (sd) 191 (64) days later. Success rates with the new device in this group were higher than those switched from Anapen to EpiPen or JEXT without device-specific training in the Device Switch Scenario – 23/36 (64%) versus 6/18 (33%) (P = 0.03) and 26/36 (72%) versus 9/18 (50%) (P = 0.11) using the 5-second time and device-specific time criteria respectively. Digital injection occurred in one (3%) participant. These success rates were not significantly different to the success rates of the same participants in their previous simulated anaphylaxis scenario using Anapen, measured by either the 5 second or device-specific criteria (data not shown).

**Table S1**. Participant characteristics at baseline, for randomised patients who did or did not complete outcome assessments at 6 weeks

|  | 6-week  Completer  (145) n (%) | 6-week  Non-completer  (13) n (%) | P |
| --- | --- | --- | --- |
| Maternal age (years) | 36.1 (6.1) | 34.8 (8.4) | 0.63 |
| Age left full time education (years) | 22.9 (5.9) | 22.2 (5.0) | 0.72 |
| Living with partner | 116 (80.0) | 5 (50.0) | 0.027 |
| Professional occupation | 70 (56.5) | 2 (22.2) | 0.079 |
| Worked in a healthcare setting | 27 (21.1) | 1 (12.5) | 1.0 |
| Non-white | 85 (59.0) | 9 (90.0) | 0.089 |
| Number of children in household: |  |  |  |
| One child | 56 (38.9) | 6 (60.0) |  |
| Two children | 57 (39.6) | 3 (30.0) |  |
| Three or more children | 31 (21.5) | 1 (10.0) | 0.40 |
| Maternal State Anxiety [STAI-1] | 36.6 (11.5) | 31.1 (8.6) | 0.096 |
| Child Age (years) | 4.1 (3.4) | 3.1 (2.2) | 0.48 |
| Male child | 89 (61.4) | 8 (61.5) | 0.99 |
| Child Weight ≥25kg | 28 (19.3) | 2 (15.4) | 1.0 |
| No food allergies | 2.9 (1.9) | 3.0 (2.0) | 0.87 |
| Eczema | 113 (81.3) | 8 (80.0) | 0.92 |
| Eczema severity [POEM] | 9.7 (7.6) | 11.3 (5.5) | 0.43 |
| Asthma | 38 (27.3) | 1 (10.0) | 0.46 |
| Partially/Uncontrolled asthma | 30 (21.6) | 1 (10.0) | 0.69 |
| Allergic rhinitis in child | 40 (29.0) | 3 (30.0) | 1.0 |
| Moderate/Severe allergic rhinitis | 37 (26.8) | 2 (20.0) | 1.0 |
| History of anaphylaxis* | 49 (33.8) | 3 (30.0) | 1.0 |
| Randomised to Epipen | 74 (51.0) | 5 (38.5) | 0.56 |
| Anaphylaxis training officer: |  |  |  |
| Researcher #1 | 23 (15.9) | 3 (25.0) |  |
| Researcher #2 | 50 (34.5) | 5 (41.7) |  |
| Researcher #3 | 72 (49.7) | 4 (33.3) | 0.51 |
| Randomised to psychological intervention | 74 (51.0) | 5 (38.5) | 0.39 |

Continuous data are presented as mean (sd).

STAI = State Trait Anxiety Inventory; POEM = Patient-Oriented Eczema Measure.

* Anaphylaxis was defined according to NIH/NIAID guidance

**Table S2**. Participant characteristics at baseline, for randomised patients who did or did not complete outcome assessments at one year

|  | One year  Completer  (110) n (%) | One year  Non-completer  (48) n (%) | P |
| --- | --- | --- | --- |
| Maternal age (years) | 36.3 (6.2) | 35.4 (6.6) | 0.42 |
| Age left full time education (years) | 23.1 (5.5) | 22.1 (6.6) | 0.11 |
| Living with partner | 92 (83.6) | 29 (64.4) | 0.009 |
| Professional occupation | 58 (61.1) | 14 (36.8) | 0.011 |
| Worked in a healthcare setting | 19 (19.6) | 9 (23.1) | 0.65 |
| Non-white | 60 (55.0) | 34 (75.6) | 0.018 |
| Number of children in household: |  |  |  |
| One child | 40 (36.7) | 22 (48.9) |  |
| Two children | 49 (45.0) | 11 (24.4) |  |
| Three or more children | 20 (18.3) | 12 (26.7) | 0.059 |
| Maternal State Anxiety [STAI-1] | 37.0 (11.0) | 34.2 (12.0) | 0.076 |
| Child Age (years) | 3.8 (3.1) | 4.6 (3.9) | 0.51 |
| Male child | 71 (64.5) | 26 (54.2) | 0.22 |
| Child Weight ≥25kg | 15 (13.6) | 15 (31.3) | 0.009 |
| No food allergies | 2.8 (1.9) | 3.2 (2.0) | 0.29 |
| Eczema | 87 (82.9) | 34 (77.3) | 0.43 |
| Eczema severity [POEM] | 9.5 (7.6) | 10.6 (7.2) | 0.39 |
| Asthma | 27 (25.7) | 12 (27.3) | 0.84 |
| Partially/Uncontrolled asthma | 20 (19.0) | 11 (25.0) | 0.41 |
| Allergic rhinitis in child | 28 (26.9) | 15 (34.1) | 0.38 |
| Moderate/Severe allergic rhinitis | 26 (25.0) | 13 (29.5) | 0.57 |
| History of anaphylaxis* | 33 (30.0) | 19 (42.2) | 0.14 |
| Randomised to Epipen | 59 (53.6) | 20 (41.7) | 0.17 |
| Anaphylaxis training officer: |  |  |  |
| Researcher #1 | 17 (15.5) | 9 (19.1) |  |
| Researcher #2 | 38 (34.5) | 17 (36.2) |  |
| Researcher #3 | 55 (50.0) | 21 (44.7) | 0.78 |
| Randomised to psychological intervention | 51 (46.4) | 28 (58.3) | 0.17 |

Continuous data are presented as mean (sd).

STAI = State Trait Anxiety Inventory; POEM = Patient-Oriented Eczema Measure.

* Anaphylaxis was defined according to NIH/NIAID guidance

**Table S3.** Six-week outcomes for randomised patients who did or did not complete a one year outcome assessment.

|  | One year  Completer  (109) n (%) | One year  Non-completer  (36) n (%) | P |
| --- | --- | --- | --- |
| Primary Outcome (6 weeks) |  |  |  |
| Successful EAI administration  (5 second criterion) | 51 (47.2) | 10 (27.8) | 0.041 |
| Secondary Outcomes (6 weeks) |  |  |  |
| Successful EAI administration (minimum discharge time) | 58 (53.7) | 14 (40.0) | 0.16 |
| Adverse events (digital injection) | 3 (2.8) | 2 (5.6) | 0.42 |
| Time device held in place (sec) | 6.9 (4.9) | 5.8 (4.8) | 0.25 |
| Post-scenario confidence (1-10)  in using EAI device | 7.6 (2.3) | 7.1 (2.9) | 0.50 |
| Area massaged after simulated injection | 59 (54.1) | 21 (58.3) | 0.66 |
| Device applied to correct anatomical position | 97 (89.0) | 29 (80.6) | 0.19 |
| Child held in correct position | 76 (69.7) | 23 (63.9) | 0.51 |
| Emergency services called | 86 (78.9) | 25 (69.4) | 0.25 |

Continuous data are presented as mean (sd).

**Table S4.** Results of imputation for missing data, for patients randomly allocated to Anapen or Epipen

|  | Anapen  (79) n (%) | Epipen  (79) n (%) | P | RR (95% CI) |
| --- | --- | --- | --- | --- |
| Primary Outcome (6 weeks) |  |  |  |  |
| Successful EAI administration  (5 second criterion) |  |  |  |  |
| ITT where missing data resulted in failure | 30 (38.0) | 31 (39.2) | 0.87 | 0.97 (0.65, 1.43) |
| ITT where missing data resulted in success | 38 (48.1) | 37 (46.8) | 0.87 | 1.03 (0.74, 1.43) |
| Secondary Outcome (6 weeks) |  |  |  |  |
| Successful EAI administration  (minimum discharge time) |  |  |  |  |
| ITT where missing data resulted in failure | 32 (40.5) | 40 (50.6) | 0.20 | 0.80 (0.57, 1.13) |
| ITT where missing data resulted in success | 40 (50.6) | 47 (59.5) | 0.26 | 0.85 (0.64, 1.13) |
| Secondary Outcomes (one year) |  |  |  |  |
| Successful EAI administration  (5 second criterion) |  |  |  |  |
| ITT where missing data resulted in failure | 28 (35.4) | 35 (44.3) | 0.26 | 0.80 (0.54, 1.18) |
| ITT where missing data resulted in success | 56 (70.9) | 55 (69.6) | 0.86 | 1.02 (0.83, 1.25) |
| Successful EAI administration  (minimum discharge time) |  |  |  |  |
| ITT where missing data resulted in failure | 30 (38.0) | 42 (53.2) | 0.055 | 0.71 (0.50, 1.01) |
| ITT where missing data resulted in success | 58 (73.4) | 62 (78.5) | 0.46 | 0.94 (0.79, 1.12) |

EAI = epinephrine autoinjector; RR = relative risk

CI = 95% confidence intervals; ITT = intention to treat

RRs are for Anapen versus Epipen

**Table S5.** Ability to use their epinephrine autoinjector in participants allocated to Anapen or Epipen – Per Protocol analysis

|  | Anapen  n (%) | Epipen  n (%) | P | OR  (95% CI) | Adj P | Adj OR  (95% CI) |
| --- | --- | --- | --- | --- | --- | --- |
| Primary Outcome (6 weeks) | (*n* = 62) | (*n* = 95) |  |  |  |  |
| Successful EAI administration  (5 second criterion) | 26 (41.9) | 49 (51.6) | 0.24 | 0.68  (0.36, 1.29) | 1.00 | 1.00  (0.45, 2.25) |
| Primary reason for failure: |  |  |  |  |  |  |
| Failed to remove all safety caps | 21 (33.9) | 20 (21.1) |  |  |  |  |
| Used incorrect end of device | 5 (8.1) | 5 (5.3) |  |  |  |  |
| Device not activated | 0 (0.0) | 3 (3.2) |  |  |  |  |
| EAI applied for < 5 seconds | 10 (16.1) | 18 (18.9) | 0.25 |  |  |  |
| Secondary Outcomes (6 weeks) |  |  |  |  |  |  |
| Successful EAI administration  (minimum discharge time) | 28 (45.2) | 62 (65.3) | 0.013 | 0.44  (0.23, 0.84) | 0.46 | 0.74  (0.33, 1.64) |
| Adverse events (digital injection) | 1 (1.6) | 4 (4.2) | 0.37 | 0.38  (0.04, 3.45) | 0.35 | 0.32  (0.03, 3.57) |
| Time device held in place (sec) | 6.1 (4.7) | 7.9 (5.2) | 0.023 | -1.80  (-3.37,-0.26) | 0.32 | -0.94  (-2.80, 0.94) |
| Post-scenario confidence (1-10)  in using EAI device | 7.5 (2.4) | 7.5 (2.5) | 0.92 | 0.02  (-0.80, 0.75) | 0.48 | 0.34  (-0.66, 1.25) |
| Area massaged after simulated injection | 36 (58.1) | 56 (58.3) | 0.97 | 0.99  (0.52, 1.89) | 0.84 | 0.92  (0.42, 2.01) |
| Device applied to correct anatomical position | 53 (85.5) | 90 (93.8) | 0.08 | 0.39  (0.13, 1.17) | 0.12 | 0.31  (0.07, 1.38) |
| Child held in correct position | 41 (66.1) | 73 (76.0) | 0.18 | 0.62  (0.30, 1.24) | 0.18 | 0.57  (0.25, 1.30) |
| Emergency services called | 52 (83.9) | 76 (79.2) | 0.46 | 1.37  (0.59, 3.16) | 0.30 | 1.71  (0.62, 4.75) |
| Secondary Outcomes (one year) | (*n* = 26) | (*n* = 88) |  |  |  |  |
| Successful EAI administration  (5 second criterion) | 15 (57.7) | 61 (69.3) | 0.27 | 0.60  (0.25, 1.49) | 0.27 | 0.52  (0.16, 1.65) |
| Primary reason for failure: |  |  |  |  |  |  |
| Failed to remove all safety caps | 9 (34.6) | 7 (8.0) |  |  |  |  |
| Used incorrect end of device | 0 (0.0) | 11 (12.5) |  |  |  |  |
| Device not activated | 0 (0.0) | 1 (1.1) |  |  |  |  |
| EAI applied for < 5 seconds | 2 (7.7) | 8 (9.1) | 0.011 |  |  |  |
| Successful EAI administration  (minimum discharge time) | 15 (57.7) | 69 (78.4) | 0.035 | 0.38  (0.15, 0.95) | 0.074 | 0.33  (0.10, 1.11) |
| Adverse events (digital injection) | 0 (0.0) | 9 (10.2) | 0.089 | - | - | - |
| Time device held in place (sec) | 7.3 (4.4) | 9.0 (4.2) | 0.14 | -1.71  (-3.53, 0.09) | 0.050 | -2.53  (-5.02, -0.02) |
| Post-scenario confidence (1-10)  in using EAI device | 7.8 (1.6) | 6.9 (2.6) | 0.22 | 0.86  (-0.45, 1.76) | 0.22 | 0.64  (-0.45, 1.71) |
| Area massaged after simulated injection | 22 (84.6) | 59 (67.0) | 0.083 | 2.70  (0.85, 8.58) | 0.075 | 4.64  (0.86, 25.14) |
| Device applied to correct anatomical position | 25 (96.2) | 86 (97.7) | 0.66 | 0.58  (0.51, 6.68) | - | - |
| Child held in correct position | 21 (80.8) | 76 (86.4) | 0.48 | 0.66  (0.21, 2.09) | 0.96 | 0.96  (0.21, 4.54) |
| Emergency services called | 23 (88.5) | 83 (95.4) | 0.20 | 0.37  (0.08, 1.77) | 0.22 | 0.27  (0.03, 2.18) |

Per-protocol population includes 42 participants allocated to receive Epipen without randomization but excludes participants who did not receive the intervention, had a 6 week visit after 90 days, or one year visit before 9 months or after 15 months.

EAI = epinephrine autoinjector

Continuous data are presented as mean (sd) and mean difference (95% CI).

Adjusted analyses controlled for participant age, baseline anxiety score, professional occupation, randomization to psychological intervention, partially/uncontrolled asthma, and training officer using logistic regression and generalized linear models.

**Table S6.** Ability to use different devices in participants trained to use Anapen or EpiPen

|  | EpiPen/Anapen  (26) n (%) | New EpiPen  (27) n (%) | JEXT  (27) n (%) | Auvi-Q  (28) n (%) | P |
| --- | --- | --- | --- | --- | --- |
| Successful AAI administration  (5 second criterion) | 4 (15.4) | 18 (66.7) | 17 (63.0) | 26 (92.9) | <0.001 |
| Successful AAI administration (minimum discharge time) | 5 (19.2) | 20 (74.1) | 20 (74.1) | 26 (92.9) | <0.001 |
| Adverse events (digital injection) | 2 (7.7) | 0 (0.0) | 1 (3.7) | 0 (0.0) | 0.26 |

**FIGURE LEGENDS**

**Figure S1**. Epinephrine auto-injector devices used in the Main Study.

**Figure S2**. Additional epinephrine auto-injector devices used in the Device Switch Study.
